# Comparison of Intravenous Anesthetic Agents for the Treatment of Refractory Status Epilepticus

**DOI:** 10.3390/jcm5050054

**Published:** 2016-05-19

**Authors:** Michael E. Reznik, Karen Berger, Jan Claassen

**Affiliations:** 1 Department of Critical Care Neurology, Columbia University Medical Center, New York, NY 10032, USA; mr3513@cumc.columbia.edu; 2 Department of Pharmacy, Weill Cornell Medical Center, New York, NY 10065, USA; kab9098@nyp.org

**Keywords:** status epilepticus, refractory status epilepticus, IV anesthetics

## Abstract

Status epilepticus that cannot be controlled with first- and second-line agents is called refractory status epilepticus (RSE), a condition that is associated with significant morbidity and mortality. Most experts agree that treatment of RSE necessitates the use of continuous infusion intravenous anesthetic drugs such as midazolam, propofol, pentobarbital, thiopental, and ketamine, each of which has its own unique characteristics. This review compares the various anesthetic agents while providing an approach to their use in adult patients, along with possible associated complications.

## 1. Introduction

Refractory status epilepticus (RSE) is defined as ongoing seizures that cannot be controlled with first- and second-line agents and has an incidence ranging from 9% to 43% [1,2,3,4,5,6]. Some patients fail to respond to third-line therapy, and are considered to have super-refractory SE (SRSE), the true incidence of which is unknown. Both of these are associated with progressively-increasing morbidity and mortality, and expert guidelines advocate early initiation of intravenous anesthetic agents to maximize the chance of seizure cessation while minimizing the risk of long-term sequelae [7]. Despite guideline recommendations, the optimal approach to management remains controversial due to a lack of evidence from high quality clinical trials. The most commonly used continuous infusion intravenous anesthetics (cIV-AEDs) include midazolam, propofol, and pentobarbital, though the use of ketamine has also been increasingly described. (Note that outside the U.S., thiopental, rather than pentobarbital, is often the barbiturate of choice, especially in Europe.) This article provides an overview of their use in adult patients and the available clinical evidence. A summary of their pharmacologic properties is provided in Table 1.

## 2. Approach to cIV Anesthetic Use

Most practitioners using cIV AEDs aim for a goal of seizure suppression or burst suppression, but even more aggressive management including suppression of all background activity has been proposed. [7] This has not been investigated in a systematic way, as the heterogeneous nature and relative rarity of the disease process makes conducting large randomized controlled trials challenging (as evidenced by the difficulty in enrolling patients in the only RCT attempted to date [7,8]). Often, total seizure suppression cannot be achieved without inducing a therapeutic coma with a burst-suppression pattern on electroencephalography (EEG) or, at times, even a completely isoelectric EEG. A meta-analysis of cIV-AEDs for RSE found that titration of treatment to EEG background suppression was associated with a significantly lower frequency of breakthrough seizures than titration to seizure suppression, but was also associated with a significantly higher frequency of hypotension; meanwhile, neither titration goal nor choice of anesthetic infusion (between propofol, midazolam, and pentobarbital) was associated with a change in overall outcome [9].

There is also no consensus on the optimal duration of anesthetic infusions for RSE, though guidelines traditionally recommend seizure control for 24–48 h, followed by a gradual wean of the infusion [7]. Seizures that occur during weaning of the anesthetic are labeled as withdrawal seizures, though there may also be later SE recurrence. Both of these necessitate resumption of the anesthetic infusion, potentially at a higher dose and/or addition of another antiepileptic. It is important to keep in mind that, while RSE carries a significant risk of poor outcome, multiple retrospective studies and case series have shown the possibility of meaningful functional recovery even when SE resolution required weeks or months, suggesting that there is no clear duration of SE or number of failures to wean IV anesthetic infusions that should be considered futile [10,11,12,13,14].

Note that there is even less agreement in treating SRSE, when cIV-AEDs fail to control seizures. The management of SRSE is outside the scope of this review, but combination therapy involves adding another treatment that is often non-pharmacologic, like hypothermia [15] or ketogenic diet [16], to ongoing treatment with cIV-AEDs. A relevant point worth noting, however, is that the use of hypothermia leads to a decrease in overall metabolism, which may lead to an increase in the half-life of the IV anesthetics referenced here.

## 3. Benzodiazepines: Midazolam

Midazolam administered as a continuous infusion has been a preferred treatment for RSE since the 1990s, with multiple case series and meta-analyses describing its successful use [9,15,16,17,18,19,20]. Its popularity stems from its favorable properties, including a fast onset (1–5 min) and relatively short half-life (in the range of 1–6 h) when used as a bolus or short-term infusion [21,22]. It also does not contain propylene glycol, unlike lorazepam and diazepam, which obviates any concern for toxicity from propylene glycol accumulation. Propylene glycol has been associated with hypotension, in addition to more severe cardiac dysfunction and metabolic acidosis, though midazolam itself can cause hypotension.

Note that although midazolam has a relatively short half-life after a single dose, case reports have demonstrated a significantly increased half-life after prolonged infusion, due to an increased free fraction and volume of distribution and accumulation of its active metabolite, leading to a longer than expected time to awakening after stopping an infusion [23,24]. Prolonged infusion can also lead to tachyphylaxis, necessitating progressively higher doses to achieve the same effect. Midazolam cIV causes respiratory depression, requiring intubation for the duration of therapy.

As with other benzodiazepines, midazolam potentiates the inhibitory action of gamma-aminobutyric acid (GABA) via binding to the gamma-subunit of the GABAA receptor [19]. It undergoes hepatic metabolism via hydroxylation from CYP 3A4 and 3A5, which forms the active metabolite 1-hydroxymidazolam that is renally excreted [25]. As a cytochrome P450 substrate, levels of midazolam are affected by AEDs and other medications that are inducers or inhibitors.

When using midazolam for RSE, a loading dose of 0.2 mg/kg at 2 mg/min is recommended, with repeated boluses of 0.2–0.4 mg/kg [7] until seizures have stopped. A continuous infusion should be started at 0.05–0.2 mg/kg/h and titrated up to 2 mg/kg/h as required, although rates as high as 2.9 mg/kg/h have been described [26]. Fernandez *et al.* compared high- and low-dose midazolam treatment protocols (if needed, as high as 2.9 mg/kg/h and 0.4 mg/kg/h, respectively) and found that the group treated under the high dose protocol had fewer withdrawal seizures after weaning off the midazolam infusion and had a significantly lower discharge mortality with no difference in hospital complications (aside from a higher incidence of hypotension, which did not affect outcome). However, though the study showed that these higher doses were probably safe, the median maximum doses in the high- and low-dose groups were 0.4 mg/kg/h and 0.2 mg/kg/h, respectively, with only half of the patients in the high-dose protocol group receiving doses higher than 0.2 mg/kg/h; meanwhile patients in the high-dose protocol also received treatment earlier, suggesting that an overall more aggressive approach to treatment may be more effective. Unfortunately, there have been no prospective trials comparing midazolam infusions to other cIV anesthetics for the treatment of RSE.

## 4. Propofol

For many, propofol is a practical alternative to midazolam as the third-line agent anesthetic of choice for RSE, chiefly because of its ultra-fast onset and rapid clearance even, in many cases, after extended infusion. Its half-life after a high-dose prolonged infusion is about 10 min for the first phase, although subsequent phases may take hours to days [27]. This property is due to the drug’s high lipid solubility, allowing it to cross the blood-brain barrier and redistribute to peripheral tissues rapidly, where it also tends to accumulate after prolonged infusions [28,29]. Its most frequent side effects are hypotension, which often requires the use of vasopressors for the higher doses that are utilized in RSE, and respiratory depression, though it may also cause bradycardia. Hypertriglyceridemia is also common given its formulation as a lipid emulsion, and significantly elevated serum triglycerides (*i.e.*, exceeding 400 mg/dL) should prompt consideration about alternative therapy. More seriously, propofol carries with it the major caveat of an uncommon but life-threatening adverse effect known as propofol-related infusion syndrome (PRIS), which was initially described in children, but has also been associated with prolonged infusions as in those used for RSE [30]. This is manifested as severe metabolic acidosis, rhabdomyolysis, renal failure, and circulatory collapse, and has been attributed to mitochondrial dysfunction that leads to a mismatch between energy supply and demand [31]. Prolonged use of propofol should, therefore, prompt routine monitoring of lactate, creatine kinase, triglycerides, and potassium. Due to the risk of PRIS and reported case reports of fatal events, propofol is not routinely recommended in pediatric patients and is contraindicated for use in young children [7].

Despite this risk, multiple case series [16,28,31,32] and a meta-analysis [9] appear to show that its complication rate and efficacy is comparable to other cIV anesthetics used for RSE. (Interestingly, there have also been some case reports about seizure-like phenomena occurring during propofol use, primarily in the anesthesia literature and especially during induction and emergence, however this appears to be a rare occurrence [33]). Again, prospective data is limited, aside from a small prospective trial comparing propofol with pentobarbital that was only able to enroll 24 patients [8].

Propofol’s mechanism of action involves modulating GABAA receptors; in addition, however, it also acts on sodium and calcium channels, and likely also has an antagonistic effect on NMDA receptors [26,34]. Its metabolism is primarily through hepatic glucuronidation with subsequent renal excretion, though a significant portion is also metabolized by cytochrome P450 isozymes. However the redistribution of propofol from blood into tissues is much more rapid than from peripheral compartments back into the blood, so clearance is also highly dependent on the total volume of distribution.

Propofol for RSE is typically given as a loading dose of 1–2 mg/kg followed by a continuous infusion, which can range from a starting rate of 20 mcg/kg/min up to as high as 200 mcg/kg/min (though infusions higher than 80 mcg/kg/min should be used cautiously due to the risk of PRIS) [7]. Propofol is formulated as a lipid emulsion and has a caloric value of 1.1 kcal/mL which should be accounted for when calculating nutritional requirements.

## 5. Barbiturates: Pentobarbital and Thiopental

Until the advent of propofol and midazolam infusions, barbiturates were the agents of choice for treating RSE. Traditionally, pentobarbital has been the barbiturate used in the U.S., while thiopental is more commonly used in Europe. In current practice, though, barbiturates are typically reserved for the management of RSE that fails to respond to midazolam and/or propofol, also known as super-refractory SE (SRSE). Though the class of medications has a long history of successful use [4,9,35,36,37,38,39,40,41,42], a host of major side effects limit its appeal given the other available options. These side effects include severe hypotension (often necessitating the use of vasopressors) and overall cardiovascular depression, respiratory depression, paralytic ileus, lowering of core body temperature, immune suppression, and a potential risk for pancreatic and hepatic dysfunction. The IV formulation of pentobarbital (but not thiopental) also contains propylene glycol, leading to a risk of propylene glycol toxicity with prolonged infusions.

In their favor, however, are several studies that suggest a possible benefit over other cIV-AEDs. A meta-analysis of 28 studies comparing midazolam, propofol, and pentobarbital infusions for RSE suggested that treatment with pentobarbital was associated with a significantly lower frequency of short-term treatment failure, breakthrough seizures, and changes to a different continuous infusion [9]. In a more recent single-center retrospective study, episodes of RSE in which barbiturates were used were associated with EEG burst suppression or complete suppression significantly more frequently than episodes in which they were not given. However they were also associated with significantly longer hospital stays for surviving patients, while mortality and likelihood of returning to clinical baseline at discharge did not differ significantly compared to propofol or midazolam [4]. Another recent single-center retrospective study confirmed these findings, and also found that weaning from pentobarbital appeared to be more successful (with lower incidence of withdrawal seizures) when phenobarbital was added before weaning [42]. Finally, a previously-mentioned, small randomized trial comparing pentobarbital with propofol showed comparable mortality and return to clinical baseline between the two, along with similar rates of infection and hypotension, though pentobarbital was associated with a significantly longer duration of mechanical ventilation [8].

Both pentobarbital and thiopental exert their effects via binding to the GABA receptor and prolonging the duration of opening of the associated chloride channel (as opposed to the increased frequency of opening caused by benzodiazepines), enhancing its inhibitory effects [43]. Since both barbiturates are highly lipophilic, they quickly distribute into the central nervous system, allowing for a fast time to onset for pentobarbital (15–20 min), and an ultra-fast time to onset for thiopental (30–60 s). However this may also result in deposition into peripheral tissues and saturation of metabolic pathways even after relatively short infusions, leading to nonlinear metabolism and a long half-life (ranging from 15 to 60 h for pentobarbital, and 11 to 36 h for thiopental) [44,45,46,47]. They both also have a tendency toward autoinduction which typically takes days to occur, as well as numerous drug interactions. Both pentobarbital and thiopental are hepatically metabolized; the main metabolite of thiopental is pentobarbital. Both pentobarbital [48] and thiopental [49] are highly protein-bound in plasma, ranging from 60% to 90%.

When used for RSE, pentobarbital is administered as a loading dose of 5–15 mg/kg, with an additional 5–10 mg/kg as needed to obtain the desired effect (infused at a rate ≤ 50 mg/min); a continuous infusion is started at a rate ranging from 0.5 to 5 mg/kg/h [7]. Thiopental is administered as a loading dose of 2–7 mg/kg (infused at a rate ≤ 50 mg/min), with additional doses of 1–2 mg/kg as needed, followed by a continuous infusion at a rate of 0.5–5 mg/kg/h. Therapeutic monitoring of pentobarbital levels may be useful for patients where brain death is considered, but should not be used to guide therapy for RSE. As mentioned in the retrospective study above, it may also be preferable to start phenobarbital in anticipation of weaning pentobarbital, to potentially reduce the risk of withdrawal seizures [42].

## 6. Ketamine

Ketamine has emerged as a more recent addition to the arsenal of cIV-AED treatment for RSE, primarily due to its alternative mechanism, though current evidence only supports its use in conjunction with other anesthetics. As opposed to the agents mentioned above, all of which act on the GABA receptor, ketamine is an antagonist on the N-methyl D-aspartate (NMDA) receptor, thereby inhibiting glutamate activity. It has a very high lipid solubility leading to a fast onset and extensive distribution, with an elimination half-life of around 2–3 h. Metabolism is primarily hepatic, with oxidation via the cytochrome P450 system (especially CYP3A4) predominantly into the active metabolite norketamine, which is then glucuronidated and excreted in urine and bile [50].

Its side effect profile is also generally favorable, as it is not associated with cardiac depression and hypotension as with the other IV anesthetics, but instead induces a positive sympathetic response, sometimes leading to drug-induced hypertension. Note, however, that in certain patients these cardiac effects may be detrimental, especially in those with coronary disease or significant cardiomyopathy. Prior studies had raised concern about the risk of increased intracranial pressure, but more recent studies show no changes in intracranial pressure with the use of ketamine [51,52]. Though it has potent anesthetic and analgesic properties, ketamine is not typically associated with respiratory depression, although hypersalivation may become an issue. Meanwhile, patients who regain consciousness after ketamine is stopped may experience psychiatric emergence phenomena, including agitation, confusion, and psychosis.

Though experience with ketamine for RSE is more limited than with the other cIV-AEDs, a number of case reports and case series detail its use [51,53,54,55,56,57,58,59,60,61,62,63]. Both a multi-center retrospective study by Gaspard *et al.* [51] and a meta-analysis that included it, along with 22 other studies [63], showed that ketamine appeared to contribute to seizure control in RSE for approximately 57% of adult patients—however, outcomes and more detailed information for most of these patients in the meta-analysis was not available. In the study by Gaspard *et al.*, ketamine was thought to be likely primarily responsible for seizure control in 32% of RSE episodes, while early response to ketamine was also associated with a significantly improved mortality rate. Of note, because ketamine is often started only after other anesthetic drugs have failed, there remains the possibility that its efficacy may be higher if introduced earlier.

There has been significant variation in reported dosing, but median loading doses appear to be in the range of 1–2 mg/kg, with a continuous infusion ranging anywhere from 1 to 10 mg/kg/h, based on the cited reports. Importantly, in almost all cases, ketamine was initially added to at least one other cIV-AED with subsequent weaning of other anesthetics; evidence is lacking as to its potential efficacy as a stand-alone cIV-AED. In the meantime, expert opinion suggests that it be preferentially used in conjunction with another anesthetic, preferably one with GABAergic action.

## 7. Controversies of Prolonged Anesthetic Use

Aside from the adverse effects listed above for individual anesthetic agents, prolonged anesthetic use comes with its own set of possible repercussions, and these have become more widely recognized. This has led to controversy about whether or not treatment of RSE with cIV anesthetic drugs may actually worsen outcomes, as suggested by several recent observational studies.

Kowalski *et al.* [64] found that anesthetic use predicted poor outcome and death in SE, with patients receiving these drugs having a three-fold relative risk of poor outcome compared to those who did not (though no attempts were made in this study to adjust for possible confounders). A study by Sutter *et al.* [65] showed that patients receiving anesthetics had four times more infections during SE and a nearly three-fold relative risk for death, despite attempts being made to account for possible confounders, such as SE duration and severity, other antiepileptic drugs used, and degree of overall critical illness. Marchi *et al.* [66] echoed these findings in a larger study that was also adjusted for possible confounders, with a subgroup analysis that also showed the association with poor outcome was strongest in patients with more benign SE subtypes (*i.e.*, absence, simple partial, or even complex partial SE). This may be deceiving, however, as such SE subtypes are rarely treated with cIV-AEDs by experienced practitioners, and their inclusion may have been responsible for any significant findings in the study.

Above all, perhaps the biggest criticism of these studies is that, even with attempts made to account for confounding factors, there was likely some degree of inherent bias in prescribing anesthetic agents to patients who were likely more ill, in ways that could not necessarily be captured by the authors’ analyses. Though they do raise valid questions about the possible harms associated with cIV-AEDs, caution should be used in interpreting these studies in such a way as to meaningfully affect clinical practice, at least until high-quality prospective evidence becomes available. Meanwhile, the general intensive care literature has raised awareness of another issue, suggesting that sedation, in general, especially in higher doses, appears to be associated with higher incidences of cognitive dysfunction, as described in a recent meta-analysis [67].

## 8. Conclusions

RSE carries with it a high morbidity and mortality regardless of treatment, though more aggressive management aimed toward early seizure cessation may improve outcomes. Multiple anesthetics have been shown to be effective in treating RSE, each with their own pros and cons but, unfortunately, there is not yet strong evidence from prospective trials to guide specific management with regards to choice of anesthetic and duration of treatment. Until such trials do exist, current clinical practice guidelines allow for flexibility in choice of anesthetic, so that the decision can be tailored to each individual case.

## Figures and Tables

**Table 1:** Pharmacology of Commonly Used CI Anesthetics for RSE.

	Mechanism of Action	Metabolism	Active Metabolite	Half-Life (Hours)	Half-Life Considerations	Drug Interactions	Examples of Drug-Drug Interactions	Adverse Reactions
Midazolam	GABA agonist	Hepatic	1-hydroxy-midazolam (renally eliminated)	2–7	Duration prolonged in renal failure and with extended duration of use	CYP 3A4 substrate	Phenytoin and phenobarbital (CYP 3A4 inducers) → lower midazolam concentrations	➢ Hypotension ➢ Respiratory depression
Propofol	GABA agonist; NMDA antagonist properties	Hepatic	N/A	0.5–7	Duration may be prolonged with extended duration of use	N/A	N/A	➢ Hypotension ➢ Respiratory depression PRIS ➢ ↑ Triglycerides
Pentobarbital	GABA agonist; Barbiturate	Hepatic	N/A	15–50	Duration may be prolonged with extended duration of use	CYP 2A6 inducer	Valproate (decreases barbiturate metabolism) → May increase pentobarbital concentrations Lamotrigine (CYP 2A6 substrate) → pentobarbital lowers lamotrigine concentrations	➢ Hypotension ➢ Respiratory depression ➢ Paralytic ileus ➢ Immune suppression ➢ Hepatic/pancreatic dysfunction ➢ ↓ Body temperature ➢ Propylene glycol toxicity
Ketamine	NMDA antagonist	Hepatic	Norketamine (hepatically eliminated)	2.5	N/A	CYP 2C9 & 3A4 substrate	Phenytoin and phenobarbital (CYP 2C9 inducers) → lower ketamine concentrations	➢ Hypertension ➢ Hypersalivation ➢ Hallucinations ➢ Emergence reaction

PRIS = propofol-related infusion syndrome.
